# Unspoken inequality: how COVID-19 has exacerbated existing vulnerabilities of asylum-seekers, refugees, and undocumented migrants in South Africa

**DOI:** 10.1186/s12939-020-01259-4

**Published:** 2020-08-20

**Authors:** Ferdinand C. Mukumbang, Anthony N. Ambe, Babatope O. Adebiyi

**Affiliations:** 1 Burden of Disease Research Unit, South African Medical Research Council, Cape Town, South Africa; 2 Department of History, University of the Western Cape, Cape Town, South Africa; 3 Child and Family Unit/Social work, University of the Western Cape, Cape Town, South Africa

**Keywords:** COVID-19, Asylum-seekers, Refugees, Undocumented migrants, Mental health, South Africa

## Abstract

An estimated 2 million foreign-born migrants of working age (15–64) were living in South Africa (SA) in 2017. Structural and practical xenophobia has driven asylum-seekers, refugees, and undocumented migrants in SA to abject poverty and misery. The Coronavirus Disease 2019 (COVID-19) containment measures adopted by the SA government through the lockdown of the nation have tremendously deepened the unequal treatment of asylum-seekers and refugees in SA. This can be seen through the South African government’s lack of consideration of this marginalized population in economic, poverty, and hunger alleviation schemes. Leaving this category of our society out of the national response safety nets may lead to negative coping strategies causing mental health issues and secondary health concerns. An effective response to the socioeconomic challenges imposed by the COVID-19 pandemic should consider the economic and health impact of the pandemic on asylum-seekers, refugees, and undocumented migrants.

## Résumé

On estime que 2 millions de migrants internationaux en âge de travailler (15–64 ans) vivaient en Afrique du Sud en 2017. La xénophobie structurelle et pratique a conduit les demandeurs d’asile, les réfugiés et les migrants sans papiers en Afrique du Sud à la pauvreté et à la misère. Les mesures de confinement de la maladie coronavirus 2019 (COVID-19) adoptées par le gouvernement sud-africain à travers le confinement de la nation ont considérablement approfondi le traitement inégal des demandeurs d’asile et des réfugiés en Afrique du Sud. Le gouvernement sud-africain ne tient pas compte de cette population marginalisée dans les programmes économiques, de lutte contre la pauvreté et de lutte contre la faim. Le fait de laisser cette catégorie de notre société hors des filets de sécurité nationaux d’intervention peut entraîner des stratégies d’adaptation négatives qui causent des problèmes de santé mentale et des problèmes de santé secondaires. Une réponse efficace aux défis socioéconomiques imposés par la pandémie du COVID-19 devrait tenir compte de l’impact économique et sanitaire de la pandémie sur les demandeurs d’asile, les réfugiés et les migrants sans papiers.

## Background

South Africa (SA) has been a preferred destination for many migrants from other parts of Africa, particularly those from the Southern African Development Community (SADC) countries. An estimated 2 million foreign-born migrants of working age (15–64) were living in SA in 2017, representing 5.3% of the South African labor force. Between 2012 and 2017, there was a 1.4% increase in international migrants of working age in SA [1]. Unofficially, the foreign-born migrant population in SA today is estimated to be around 4.2 million [2].

SA’s commitment to upholding human rights and the rights of asylum-seekers and refugees make the country an attractive destination for people fleeing their home countries in the quest for a more dignifying and humane existence. The economy of SA, which is one of the most advanced on the African continent, has contributed to the exponential increase in the number of people seeking asylum from the continent and the world at large.

The unprecedented flow of asylum-seekers and refugees into SA has compromised the government’s stance to adhere to its commitment towards upholding human rights while delivering its promise to uplift the socio-economic welfare of its citizens; especially those that still feel the brunt of apartheid – a system of institutionalized racial segregation. While there is the political will to accommodate and cater for asylum-seekers, refugees, and undocumented migrants in SA, the increasing economic and financial woes of the country has led to the government adopting and frequently changing laws that in many ways, have impacted negatively on the lives of these foreign-born migrants.

The South African Refugees Act provides the right for asylum-seekers and refugees to work and study, to access medical services and life-saving treatment and freedom of movement [3]. SA’s considerate assertion to these rights partly accounts for the influx of asylum-seekers and refugees. However, failure to regularize the national asylum system, bureaucratic inefficiency, and corruption have engendered issues such as lack of personnel capacity and logistics to deal with the volume of asylum-seekers and refugees, consequently, creating a backlog in the processing and adjudication of the documents [3–5]. To this end, many foreign-born migrants remain undocumented and/or asylum-seekers for years, and those with refugee status find it difficult to obtain documents like the refugee identity or travel document [4].

The difficulty in obtaining or renewing documents on time makes it challenging for most asylum-seekers and refugees and, of course, impossible for undocumented migrants to gain any meaningful and long-term employment even if they are qualified. Most of them are relegated to the informal sector and nudged to reside in underprivileged communities. Foreign-born migrants are, therefore, more likely to be informally employed and face precarious employment conditions [1]. According to the African Centre for Migration and Society [1], a foreign-born migrant with the same age, gender, and level of education, belonging to the same ‘population group’ and living in the same place as a South African, has a higher probability of being employed than a South African. Therefore, there is the conception in most communities where these foreign-born migrants reside and work that they deprive South Africans of employment and other business opportunities and are a strain on the limited social services and amenities, constituting the main drivers of xenophobia [6]. To avoid the killing of foreign-born migrants by the general population, the South African government enacted bylaws that make it challenging for foreign-born migrants to gain employment in SA. The abovementioned structural and practical xenophobia have plunged foreign-born migrants living in SA into abject poverty and misery [6, 7].

Most asylum-seekers, refugees, and undocumented migrants entering SA come from regions with endemic malaria, HIV, and TB infections [8–10]. In addition to these infections, there is equally a huge burden of non-communicable diseases among foreign-born migrants living in SA [11]. Migration also involves going through certain stages involving lack of preparedness, difficulties in adjusting to the new environment, the complexity of the local system, language difficulties, cultural disparities and adverse experiences, which can cause distress and anxiety to the foreign-born migrants with a negative impact on their mental well-being [12]. Despite this high disease burden, foreign-born migrants face various challenges accessing preventative and curative healthcare services including the lack of migration-aware and mobility-competent health systems programs [11].

## Main text

### Unspoken inequality

**Fig. 1:**
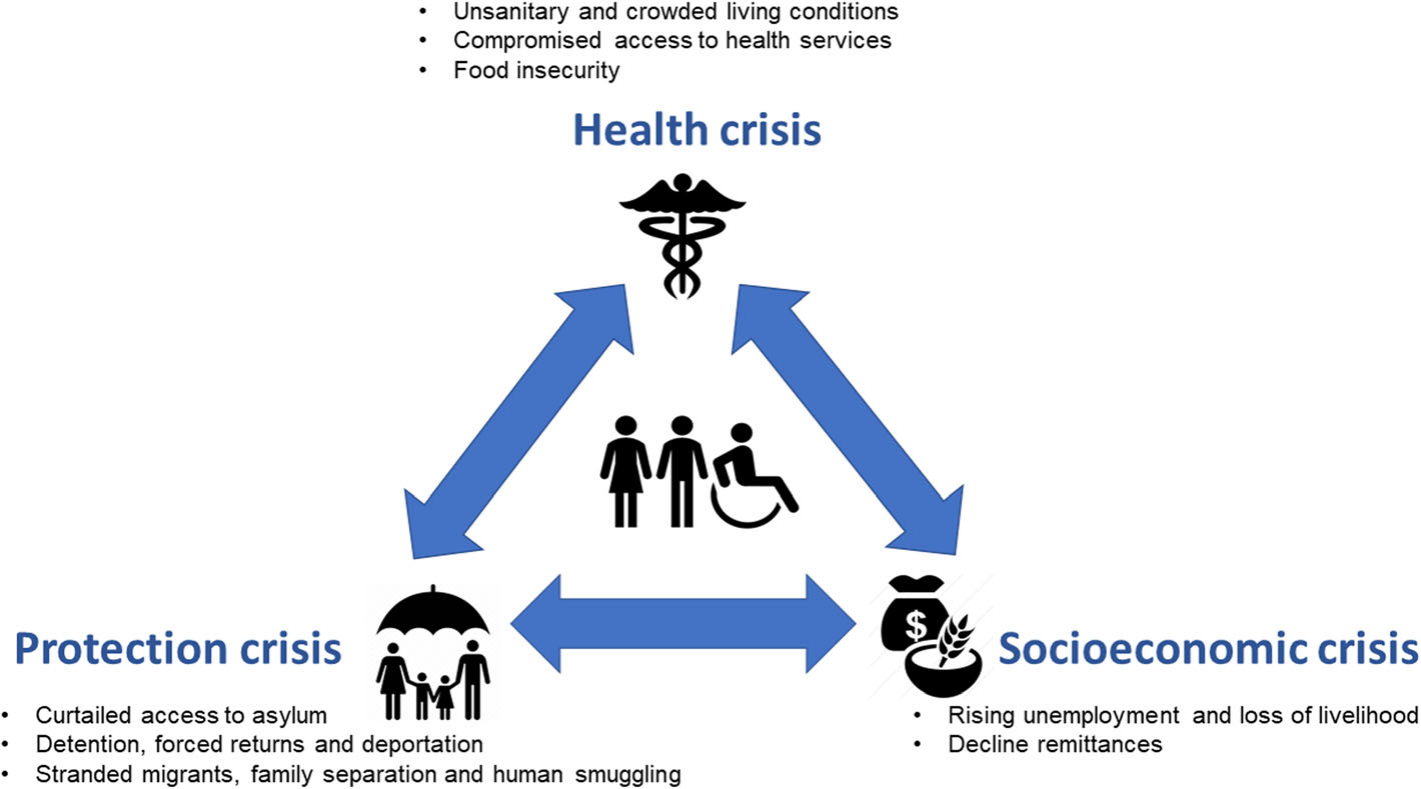
Crisis impacting foreign-born migrants – Adapted from United Nations [17]

On the 23 of March 2020, President Cyril Ramaphosa announced a nation-wide lockdown to help curb the spread of the Coronavirus Disease 2019 (COVID-19) epidemic in SA [13] and to enable the health systems to prepare for the increasing influx of moderate to severe COVID-19 cases [14]. In addition to the national lockdown, other social distancing measures such as isolation of individuals infected with the SARS-CoV-2 virus and quarantining of those who may have been exposed to or were in contact with an infected person are also encouraged or enforced [15]. In spite of these containment measures, SA has the highest number of infections with SARS-CoV-2 in Africa with  more than a 100,000 cases and 2000 deaths reported as of the middle of June 2020 [16]. While these containment measures are estimated to have negatively affected all those living in SA, asylum-seekers, refugees, and undocumented migrants disproportionally experience the negative impacts of the pandemic because of existing vulnerabilities affecting this population Fig. 1 [17].

Before the lockdown containment measures, foreign-born migrants living in SA had relatively weakened social support structures, bleak socio-economic prospects, unequal access to health care and social services, precarious housing conditions, tenuous living and working conditions, and higher risks of exploitation and abuse [18]. The lockdown containment measures worsened their conditions as they found themselves suddenly jobless, being evicted from their homes, hunger insecure, and trapped in dormitories or camps where adequate physical distancing is impossible [19]. These conditions have created and continue to fuel feelings of uncertainty, distress, anxiety, fear, anger, and hopelessness; conditions considered as precursors and prodromes of mental health illnesses such as anxiety and depression [20, 21], and secondary health concerns – neglect of self-care, respiratory infections, HIV, and substance abuse [22]. Studies conducted in other countries have demonstrated that nation-wide lockdown is associated with depressive symptoms, anxiety symptoms, and sleeping disturbances [23, 24].

Organizations working with foreign-born migrants during the COVID-19 pandemic containment measures have raised concerns regarding the arrest and detention of foreign-born migrants [25], their placement in, and subsequent repatriation from camps and shelters. There are also reports that foreign-born migrants are more likely to be arrested for minor offenses during the lockdown period and less likely to be released on bail because of expired documentation [25]. The closure of the Department of Home Affairs, which is responsible for renewing and issuing refugee permits, asylum permits, and residence permits, has made many foreign-born migrants vulnerable to harassment and extortion by law enforcement agents who are likely to ignore the moratorium on arrests of all those whose permits expired during the lockdown [2]. Migrants are consequently less willing to seek testing or care for COVID-19 symptoms as they are afraid of being detained or deported. These repatriation centers and prison stations are also prone to overcrowding, making it challenging to practice social distancing and recommended hygiene measures [26]. Under such conditions, these foreign-born migrants are at heightened risks of contracting COVID-19 but tend not to seek care when they notice the signs and symptoms of COVID-19, which makes them more likely to spread or die from the disease.

During the mandatory nationwide lockdown, asylum seekers, refugees, and undocumented migrants found themselves trapped indoors leading to the feeling of isolation [27]. Isolation, scarcity of resources, and the lack of social contacts may have created and continue to fuel a negative impact on people’s emotions and psychological well-being [28, 29]. Also, with an increasing number of cases in SA, there has been a significant increase in the stigmatization of anyone remotely related to COVID-19. Stigma and discrimination that stems from COVID-19 can occur when people associate it with nationality or being a foreign-born migrants and can lead to further social avoidance, denial of health care, and perhaps even violence fueling secondary health concerns [30]. Stigma also makes people feel isolated; even to the point of feeling abandoned and increases the chances of anxiety and depression [31].

Although lockdown is comparatively easier for people who live in houses with big gardens, it is harder for those who live in crowded homes and camps, sharing their living spaces and toilets with non-relatives and strangers such as is the condition of most asylum-seekers, refugees, and undocumented migrants living in SA [32]. Under these conditions, the risk of abuse (emotional and sexual), exploitation, and violence are heightened. Evidence shows that foreign-born migrants living under such circumstances in sub-Saharan Africa have consistently poorer physical and mental health outcomes than others [33].

To address some of the socio-economic hardship that the COVID-19 pandemic containment measures have placed on the South African population, the South African government adopted various economic and hunger alleviation measures. First, the government announced the COVID-19 Social Relief of Distress grant of R350 ($20) to all South Africans who are unemployed including those who lost their job as a result of the COVID-19 pandemic for a period of 6 months from May 2020. Secondly, the South African government increased the value of the child and social support grants until October 2020. Thirdly, the government pledged a Business Relief Fund of R500 million ($30 million) for businesses that may have their operations affected by the COVID-19 pandemic. Finally, the government is providing tax subsidies for small businesses and individuals and lowering contributions to the Unemployment Insurance Fund (UIF).

While these strategies are commendable, it is unclear how asylum-seekers, refugees, and undocumented migrants living in similar or worse situations are being considered. Not only are they no longer able to acquire finances through work of any kind, but they also are not being considered in any of the government’s plans to mitigate the impact of the lockdown measures. For instance, the African Centre for Migration and Society [1] report indicates that foreign-born migrants of working age living in SA are more likely to own a business and be employers. Unfortunately, most of these businesses owned by asylum-seekers, refugees, and undocumented migrants, although their operations are equally affected by the COVID-19 crisis, are not considered for the Business Relief Fund as they are automatically excluded based on the qualification criteria – businesses must be 100% South African owned, at least 70% of employees must be South Africans and recipients must be tax compliant [34].

For asylum-seekers and special-permit holders who were employed in the formal sector and who paid the mandatory taxes into unemployment before the lockdown measures were imposed, their UIF payments are not being paid while the South African employees in the same companies receive theirs [35]. The argument for non-payment of foreign-born migrants' UIF is that the electronic system used by the UIF does not recognize foreign passport numbers [35]. The Department of Employment and Labour made these payments to help individuals cope with the worst effects of the national lockdown. For the South African citizens, the UIF provided some relief, as this income replacement allowed them to take care of their families. However, the socio-economic situation of refugees, asylum-seekers and special-permit holders not receiving the UIF has deteriorated further.

The South African government also embarked on providing food parcels to those who are threatened by food insecurity. During these food distribution campaigns, it has been observed that foreign-born migrants form a significant number of attendees [36]. Similar to accessing the Social Relief of Distress grant of R350 ($20) and receiving UIF payments, a South African national ID or special permit is required to receive food parcels, which foreign-born migrants are unlikely to possess. [36]. In this way, asylum seekers, refugees, and undocumented migrants are excluded from the government’s food relief programs [37].

The South African Department of Home Affairs announced they will ensure that anyone whose immigration status permit expires before the end of the lockdown period will not be penalized as long as they present themselves to a refugee reception office within 30 days of the lockdown ending. Nevertheless, some asylum-seekers have been undocumented for months and even years prior to the national lockdown [4]. These undocumented migrants are most vulnerable to harm and infection with COVID-19 as they are most likely to be homeless and their access to basic human rights and services is particularly limited. Consequently, the lockdown containment measures can potentially increase their vulnerabilities regarding mental health and secondary health concerns as people who are homeless are already prone to mental health issues and problems in physical health [12] due to neglect of self-care, leading to the prevalence of respiratory infections, HIV, and substance abuse.

There is an overall poor engagement of SA’s public healthcare system with migrants, therefore, testing and treatment responses within public health systems fail to engage with asylum-seekers, refugees, and undocumented migrants [38]. As discussed earlier, undocumented migrants are also usually reluctant to seek medical and other assistance due to their lack of documentation for fear of possible arrest, detention, and even deportation. According to Kruger [39], where someone suspected of having symptoms related to the SARS-CoV-2 infection refuses to take a test, an enforcement officer should apply to a magistrate for a warrant to compel such testing. Unfortunately, this cannot be applied to undocumented migrants as individuals are required to provide information on their ‘nationality’ and identification details to take a SARS-CoV-2 test in most parts in South Africa [2]. Therefore, early detection, testing, diagnosis, contact tracing, and seeking care for Covid-19 becomes challenging for undocumented migrants, thus increasing the risk of outbreaks among migrants and the general population, as COVID-19 is associated with clusters and outbreaks [14]. Their exclusion from accessing testing, treatment, and the palliative measures put in place during this COVID-19 period will, ultimately, undermine the government’s efforts to curb the spread of the virus.

### Call to action

Some civil societies such as the Scalabrini Centre of Cape Town are working to address the conditions of asylum-seekers and migrants. The Scalabrini Centre of Cape Town is a non-governmental organization based in Cape Town, SA that provides specialized services for refugee, migrant, and South African communities. For instance, the Centre has, in coordination with the major banks of SA, managed to not freeze the bank accounts of asylum-seekers and refugees whose permits got expired during the period of the national lockdown. This is meant to permit them to have access to their finances to buy food and purchase other essential goods and services. Unfortunately, many foreign-born migrants with expired permits report that their accounts are still frozen. Also, the Scalabrini Centre of Cape Town has recently won a Court Order that may ensure some of SA’s asylum-seekers and special-permit holders to be able to apply for the COVID-19 Social Relief of Distress grant. Other non-governmental organizations have focused on hunger alleviation by providing food parcels.

While the efforts made by these civil societies to alleviate hunger and provide some relief to the economic situation of asylum-seekers, refugees, and undocumented migrants are commendable, these efforts are not sustainable without the government’s support. According to the United Nations [17], four basic tenets should drive efforts to support foreign-born migrants in the era of COVID-19 and moving forward: (1) Their exclusion is costly in the long-run whereas their inclusion pays off for everyone. (2) The response to COVID-19 and protecting the human rights of migrants are not mutually exclusive. (3) No-one is safe until everyone is safe. (4) Migrants are part of the solution. The International Labour Organization (ILO) also confirms that the inclusion of foreign-born migrants in national COVID-19 policy responses can help to ensure the realization of equality and social justice [40]. Equality and social justice could be achieved by engaging with and including migrant-led organizations, civil societies, international organizations, and researchers working with migrant groups towards developing programs that consider migrants.

ILO also suggests that governments should include asylum-seekers, refugees, and undocumented migrants in their national income and related policy responses. To achieve this, foreign-born migrants living in SA will require their status in SA to be legalized as many forms of access are contingent upon having proper documentation, such as access to healthcare, education, food parcels, banking services, unemployment benefits, social grants, or even, at times, freedom of movement. The COVID-19 pandemic and its implications necessitate an inclusive approach, which leaves no one behind because our individual and collective wellbeing is precariously interconnected. In the era of the COVID-19 pandemic, a narrow citizenship approach will be inadequate in dealing with an intertwined sociality.

The South African government should also extend access to health services and social protection coverage to this category of our society [40]. For instance, provide healthcare to foreign-born migrants irrespective of migration status. The International Organization for Migration [21] also reiterates its call to ensure that foreign-born migrants and displaced persons are included in governments’ plans for mental and psychosocial support provision in the context of COVID-19 and moving forward.

## Conclusion

The response to COVID-19 and ensuring the health and well-being of asylum-seekers, refugees, and undocumented migrants are not mutually exclusive. The first step to effectively address the socioeconomic and psychosocial impact of the COVID-19 pandemic and lockdown on foreign-born migrants while reducing inequities between them and local communities involves developing policies that take their realities into account. Although these migration-aware and mobility-competent policies do not necessarily translate in practice, the South African government should engage with and include migrant-led organizations, civil society, international organizations, and researchers working with migrant groups in the development of appropriate responses. Leaving this category of our society out of the national response safety nets may engender negative coping strategies leading to mental health issues, and secondary health concerns.

## Data Availability

Data sharing is not applicable to this article as no datasets were generated or analyzed during the current study.

## Abbreviations

COVID-19: Coronavirus Disease 2019
ILO: International Labour Organization
SA: South Africa
SADC: Southern African Development Community
UIF: Unemployment Insurance Fund
